# Chemotherapy-induced peripheral neuropathy: an update on the current understanding

**DOI:** 10.12688/f1000research.8053.1

**Published:** 2016-06-22

**Authors:** James Addington, Miriam Freimer

**Affiliations:** 1 Department of Neurology, The Ohio State University, Columbus, OH, USA

**Keywords:** peripheral neuropathy, Chemotherapy-induced peripheral neuropathy, CIPN, Taxanes, Platinum agents, Vinca alkaloids

## Abstract

Chemotherapy-induced peripheral neuropathy is a common side effect of selected chemotherapeutic agents. Previous work has suggested that patients often under report the symptoms of chemotherapy-induced peripheral neuropathy and physicians fail to recognize the presence of such symptoms in a timely fashion. The precise pathophysiology that underlies chemotherapy-induced peripheral neuropathy, in both the acute and the chronic phase, remains complex and appears to be medication specific. Recent work has begun to demonstrate and further clarify potential pathophysiological processes that predispose and, ultimately, lead to the development of chemotherapy-induced peripheral neuropathy. There is increasing evidence that the pathway to neuropathy varies with each agent. With a clearer understanding of how these agents affect the peripheral nervous system, more targeted treatments can be developed in order to optimize treatment and prevent long-term side effects.

## Introduction

With the advancement of better cancer therapies, including targeted chemotherapeutic agents, come longer patient survival times and the potential for long-term treatment-related side effects. Chemotherapy-induced peripheral neuropathy (CIPN) can be a severe side effect often associated with several chemotherapeutic agents including the platinum agents, taxanes, vinca alkaloids, thalidomide, and bortezomib. CIPN is often dose dependent and progressive while receiving and after such treatment ^1– 3^. In severe cases, the pain, sensory changes, and weakness associated with CIPN can lead to dose reductions, changes in chemotherapy protocols, or termination of a therapeutic agent. The morbidity associated with CIPN can lead to pronounced alterations in quality of life and independent performance of activities of daily living ^4, 5^. Unfortunately, the specific pathogenesis and pathophysiological effects of specific agents are poorly understood ^4, 6^. A recent meta-analysis of more than 4000 chemotherapy-treated patients found the prevalence of CIPN to be 68.1% within the first month of chemotherapy treatment, 60.0% at 3 months, and 30.0% at 6 months ^6^. With such a high prevalence of disease, better understanding of the pathophysiology, early recognition of those at risk, and implementation of potential treatments are crucial to the prevention and management of CIPN. This review aims to focus on an update of the underlying pathophysiology leading to CIPN and will briefly discuss newer treatment trials and updates. As the purpose of this review is to update readers on more recent advancements, previously known work will be only briefly reviewed for familiarity and new theories will be explored in more detail.

## Pathophysiology/diagnostics

The manner in which selected chemotherapeutic agents result in CIPN has been extensively studied for many years. Numerous studies have demonstrated treatment-specific mechanisms by which such agents affect the peripheral nervous system. Disrupted microtubule-mediated axonal transport, axonal degeneration, direct damage to the dorsal root ganglion, and mitochondrial dysfunction have all been shown in previous studies. Krøigård *et al.* showed that although patients treated with oxaliplatin or docetaxel may have purely sensory symptoms and signs, electrodiagnostic testing may demonstrate both motor and sensory involvement. Additionally, a cohort of patients may have sensory symptoms yet have normal nerve conduction studies. Further evaluation with skin biopsy and/or quantitative sensory testing may suggest predominately small-fiber neuropathy ^7^. Laser Doppler imaging, a newer technique used to evaluate small-fiber neuropathies, has been shown to be more specific in terms of evaluating small-fiber dysfunction in CIPN patients ^8^. High-resolution ultrasound is another recently introduced technology to evaluate peripheral neuropathies. In non-CIPN axonal neuropathies, the cross-sectional area of a visualized nerve on ultrasound is typically reduced in comparison to compressive neuropathies (i.e. median neuropathy at the wrist, ulnar neuropathy at the elbow). Interestingly, in patients with no history of compressive neuropathies but with axonal neuropathies by nerve conduction studies presumed secondary to chemotherapy, the cross-sectional area of visualized nerves is increased.

Although the neuropathy is felt to be axonal in CIPN, this raises questions as to why the cross-sectional area is increased and whether these patients are more prone to compressive neuropathies ^9^.

## Platinum agents

The platinum agents, more specifically carboplatin and oxaliplatin, exert their anti-neoplastic effects by forming platinum-DNA adducts that ultimately lead to cell cycle arrest and apoptosis. Used for the treatment of many solid malignancies, the platinum-based agents have been associated with severe side effects, including, but not limited to, nephrotoxicity and neurotoxicity. The dorsal root ganglion is not protected by the blood-brain barrier, making the DNA within the cell body of the dorsal root ganglion preferentially susceptible to toxic agents, such as the platinum agents ^10, 11^. As a result of dorsal root ganglion damage, the neurotoxicity associated with the platinum agents presents as a sensory neuronopathy with anterograde axonal degeneration.

The platinum agent oxaliplatin is known to cause a rapid, sensory neuropathy, with some studies reporting upwards of 90% of patients being affected ^12^. The chronic neuropathy associated with oxaliplatin is present in approximately 10% of patients two years post-administration ^13^. Electrophysiologic studies have also shown persistent low sensory amplitudes out of proportion to motor studies in chronic versus acute oxaliplatin-associated neuropathy ^14^.

In addition to alterations at the DNA level, the platinum agents have long been felt to exert some of their neurotoxicity through alterations in transmembrane receptors and channels. Recent work has been instrumental in further elucidating these changes. Specifically, some of the neurotoxicity associated with oxaliplatin is presumed secondary to an underlying channelopathy. Oxalate, a metabolite of oxaliplatin, is known to prolong the open phase of voltage-gated Na ^+^ channels, leading to prolonged depolarization and nerve hyperexcitability ^15^.

Another more recent development in terms of CIPN pathophysiology relates to the functioning of transient receptor potential (TRP) channels, which are also affected by the platinum agents. The TRP channels are non-selective cation channels activated by such things as heat, acidic environments, and capsaicin. Recent work has shown that patients treated with paclitaxel or bortezomib (discussed later) showed upregulation of the TRPV1 subtype in the dorsal root ganglion ^16^. Furthermore, treatment of pain with TRPV1 antagonists in some rat models of CIPN has been successful ^17^. An additional mechanism of neuropathy of the platinum agents may involve certain membrane transporters. Both copper and organic cation transporters have been shown to facilitate the transport of carboplatin into the dorsal root ganglion of sensory nerves. In cell lines overexpressing the *CTR1* gene, carboplatin uptake is higher and preferentially localizes to the dorsal root ganglion, not the linked axon, supporting the clinical phenotype of a sensory neuronopathy ^18^. Studies have also shown that the organic cation transporter (OCT) class, specifically subtypes OCT1 and OCT2, are associated with the dorsal root ganglion and that mRNA levels are affected by the administration of carboplatin ^19, 20^.

## Taxanes

The taxane class of chemotherapeutic agents exerts its anti-neoplastic effects on the microtubule during the cell cycle. Loss of depolymerization of the microtubule leads to mitotic arrest during the G2/M phase of the cell cycle ^21^. The microtubule plays several crucial functions in maintaining the integrity and health of properly functioning axons. Patients receiving one of the taxanes often develop a length-dependent sensory neuropathy, as supported by pathological specimens demonstrating axonal degeneration and secondary demyelination in a length-dependent manner ^22^. In addition to microtubule stabilization secondary to prevention of depolymerization, previous studies have also shown mitochondrial damage in those treated with paclitaxel ^23^. Studies completed in 2011 showed that deficits in complexes I and II were seen in the sciatic nerves of those treated with paclitaxel. In rat model studies, treatment with acetyl-L-carnitine has been shown to improve the peripheral neuropathy, supporting the theory of mitotoxicity as a potential contributing factor ^24^. Lastly, studies have also shown that TRP upregulation in the dorsal root ganglion leads to neuropathic pain, as mentioned previously. Increased expression of TRPV4 has been demonstrated in the dorsal root ganglion of those treated with paclitaxel. TRPV4 knockout mouse studies have demonstrated an improvement in the neuropathic pain ^25^.

More recently, studies in zebrafish have shown that changes in the epidermis may account for some of the clinicopathological changes that occur. Paclitaxel-treated zebrafish demonstrate increases in epidermal matrix-metalloproteinase-13. Treating them with corresponding inhibitors improved their response to noxious stimuli. Similarly, fluorescein-labeled paclitaxel accumulated in the basal keratinocytes of zebrafish treated with paclitaxel, while the corresponding uptake in axons was less. This suggests that changes at the epidermal level may contribute to the underlying pathophysiology of the painful neuropathy associated with paclitaxel ^26^. Furthermore, studies in rats have shown increases in macrophages within the dorsal root ganglion. Increases in Toll-like receptor 4 leads to increased expression of monocyte chemotactic protein 1, ultimately leading to macrophage infiltration. Administration of clodronate depleted macrophage infiltration at the level of the dorsal root ganglion, improving the rats’ pain tolerance. Similarly, administering antagonists to both Toll-like receptor 4 and monocyte chemotactic protein 1 blocked the hypersensitivity experienced by the rats. Collectively, this study suggested that administration of paclitaxel to rats leads to increased Toll-like receptor 4 expression and upregulation of monocyte chemotactic protein 1 at the level of the dorsal root ganglion. Subsequently, increased macrophage infiltration leads to the production of inflammatory mediators. Blocking anywhere along this pathway improves the hypersensitivity ^27^.

In a recent prospective trial of patients treated with adjuvant oxaliplatin (for colon cancer) or docetaxel (for high-risk breast cancer), the incidence of significant neuropathy differed between the two groups. The incidence of cold-associated allodynia was almost non-existent in the docetaxel-treated patients in comparison to their oxaliplatin counterparts. Although neuropathic symptoms were present in both treatment groups, the severity was much worse in the oxaliplatin cohort. At one-year follow up, 63.6% of patients treated with oxaliplatin met diagnostic criteria for CIPN, while only 44.8% did so in the docetaxel group ^28^.

## Vinca alkaloids

The vinca alkaloid class, most commonly vincristine, can also lead to a painful peripheral neuropathy. Similar to the taxane class, the vinca alkaloids exert their anti-neoplastic effect on the microtubule during the cell cycle, ultimately leading to cell cycle arrest. Although this is the aim for cancer cells, undifferentiated effects at the peripheral nervous system can lead to unwanted side effects. Unlike the peripheral neuropathy seen with the taxanes, the vinca alkaloids often cause sensory and motor neuropathy ^29^. Vinca alkaloids have a high affinity for a-tubulin of the microtubules, affecting their assembly and ultimately leading to cell death ^30^. Recent studies in models of vincristine-induced allodynia have shown a decreased level of endomorphin-2, which is primarily found in the spinal cord and exerts its analgesic effect on mu-opioid receptors. It is proposed that such changes may lead to the hypersensitivity and allodynia experienced by patients ^31^. Furthermore, increased serine protease, which inactivates the endomorphins, was also found in the spinal cord. Blocking the serine protease pathway with diprotin A blocked the downregulation of endomorphin-2. Other studies have shown that reactive oxygen species, often produced by chemotherapy, affect serine protease activity ^32^. Afferent pain pathways also appear to be affected, as levels of c-Fos, a pre-synaptic marker, are unregulated at the spinal cord level. This suggests functional alterations along afferent pain pathways from the peripheral to central nervous system ^33^. Furthermore, piccolo, a molecule crucial for the maintenance of synaptic plasticity, is increased in both intermediate (III-IV) and superficial (I-II) laminae, suggesting the possibility of enhanced hyperactivity ^34^. **

## Thalidomide

Thalidomide was introduced for the treatment of multiple myeloma in 1999. Since that time, it has become standard of care in many protocols and has been utilized in other inflammatory conditions such as inflammatory bowel disease (i.e. Crohn’s disease). The effects of thalidomide are pleiotropic, making its specific mechanism of action leading to neurotoxicity unclear. The peripheral neuropathy associated with thalidomide treatment is often sensory or sensorimotor in nature ^35^. This usually begins within a few months of treatment onset but may persist after cessation of the agent ^36^. Interestingly, cases of autonomic dysfunction, manifesting as both orthostatic hypotension and symptomatic bradycardia, have also been described with thalidomide treatment ^37^. One clear mechanism of action of thalidomide is through its anti-angiogenic properties. It has been proposed that thalidomide may affect neuronal survival secondary to microvascular changes as a result of its anti-angiogenic properties. Additionally, it has been shown to downregulate tumor necrosis factor alpha (TNFα), ultimately leading to inhibition of nuclear factor kappa beta (NFκβ). Inhibition of NFκβ has been previously shown to lead to neuronal death, another proposed mechanism for the neurotoxic effects of thalidomide and its potential role in the development of peripheral neuropathy. More recently, in 2007, lenalidomide, a structural analog to thalidomide, was studied in the management of multiple myeloma. Although lenalidomide comes with its own side effect profile, less than 10% of patients treated developed a severe, grade 3–4, neuropathy. In addition, many of the patients in the study were previously treated with thalidomide and their peripheral neuropathy did not worsen during the course of treatment with lenalidomide ^38^.

## Bortezomib

Bortezomib is a more recent addition to cancer chemotherapeutics approved by the FDA in 2003 for the treatment of advanced myeloma. Bortezomib exerts its mechanism of action by inhibiting the 26S ribosome subunit and preventing protein degradation, leading to cell cycle arrest and apoptosis ^39^. In patients treated with bortezomib, a severe, painful, sensory neuropathy commonly develops ^40, 41^. Bortezomib has been shown to affect polymerization of α-tubulin and result in microtubule stabilization, similar to that of the taxane class ^42^. Mitochondria in the dorsal root ganglia of treated patients have shown vacuolation, presumed secondary to mitochondrial enlargement, which leads to activation of pro-apoptotic pathways ^43^. Inflammation and oxidation stress have also been implicated in the development of neuropathic pain associated with the administration of bortezomib. Inhibition of NFκB by bortezomib leads to increased TNFα and the production of reactive oxygen species, both of which have been associated with neuropathic pain ^44, 45^. Another mechanism of neuropathic pain in patients treated with bortezomib is activation of TRP channels. As mentioned earlier, increased levels of TRPV1 in the dorsal root ganglion of patients treated with bortezomib have been felt to be causative of some of the neuropathic pain appreciated by patients ^17^.

## Risk factors

The ability to predict how patients will respond or be adversely affected by specific therapies or interventions has a meaningful impact on the utility of medical treatment. An understanding of how the pre-morbid health status may impact response and side effects of chemotherapy is essential. Several risk factors have been identified which may predispose the patient to neuropathy, and diabetes mellitus is the most important. A retrospective study of 374 patients treated with taxane-based chemotherapy regimens identified 81 individuals (21.6%) as having diabetes mellitus at the time of treatment. In those diagnosed with diabetes mellitus for more than five years, the incidence of neuropathy was 75% over the course of treatment, in comparison to 48.8% and 52.8% for non-diabetics and those diagnosed for less than five years, respectively ^46^. Although this study suggests that upwards of half of those treated with taxane-based chemotherapy will develop a neuropathy, the percentage significantly increases in those with a longer-standing history of diabetes mellitus, irrespective of their current control status. In addition to pre-morbid conditions, genetic susceptibility has been investigated in recent years. Pharmacogenomics, or more appropriately toxicogenomics, is an area of science that attempts to describe genetic predispositions to developing specific conditions. For example, under homeostatic conditions, *CEP72* is involved in the proper functioning and maintenance of microtubules. In children diagnosed with acute lymphoblastic leukemia and treated with vincristine, those with single nucleotide polymorphisms affecting the promoter region of the *CEP72* gene were more likely to develop a grade 2–4 neuropathy during the course of their treatment ^47^.

## Treatment

Effective management for CIPN is lacking and therefore treatment options are limited. A 2015 publication by Majithia *et al*. reviewed several clinical trials sponsored by the National Cancer Institute aimed at addressing this void. Several of the more recent studies between 2014 and 2015 will now be reviewed. Alpha-lipoic acid (ALA) has been studied as a prophylactic agent for non-CIPNs, for example diabetic neuropathy. A randomized, double-blinded, placebo-controlled trial in patients exposed to platinum-based agents showed no statistically significant difference in those treated with ALA 600 mg three times daily versus placebo, although there was a high dropout rate amongst patients ^48^. A study of patients treated with oxaliplatin-based regimens showed no significant improvement in neuropathy outcomes in those treated with calcium and/or magnesium before and after infusions ^49^. As mentioned previously, neurotoxicity from platinum-based agents may be due to accumulation in the dorsal root ganglion. The anti-oxidant glutathione has been thought to counteract such toxicity. In a randomized trial of patients receiving carboplatin and paclitaxel, there was no significant improvement in measured outcomes in patients treated with glutathione compared to those treated with placebo ^50^.

There have been few recent studies examining the management of established CIPN. A 2014 study evaluated the combination of topical 2% ketamine plus 4% amitriptyline in patients with established CIPN, classified as taxane induced or non-taxane induced. Similar to most other studies in CIPN, there was no statistically significant difference with regard to those treated with the topical ointment versus placebo. Interestingly, those treated in the taxane-induced group reported improved symptoms, regardless of the treatment arm ^51^. In terms of CIPN pain, the selective serotonin-norepinephrine reuptake inhibitor duloxetine has shown promise. A 2013 double-blinded, placebo-controlled, crossover trial found that duloxetine 60 mg daily had a beneficial effect on CIPN-related pain ^52^. These findings were more recently confirmed in a study comparing duloxetine 40 mg daily and vitamin B12 ^53^.

## Conclusion

In conclusion, CIPN is a complex topic. No single unifying pathophysiologic process can be identified to explain the various neuropathies that occur after exposure to different chemotherapeutic agents. Although the anti-neoplastic features of these agents are well described, the neurotoxic side effects may be multifactorial and unrelated to the anti-neoplastic pathway. As we learn more about chemotherapeutic-specific neuropathic pathways, certain targeted medications, whether preventive or treatment related, may prove to be more appropriate depending on which chemotherapy agent a patient is exposed to. Although most CIPN is related to dysfunction of the peripheral nervous system, several studies have shown functional changes near and within the central nervous system, which may lead to radical changes in terms of optimal management. Additionally, the upsurge in genetics research, the field of pharmacogenomics, and tailoring each individual patient’s treatment plan based on their genetic predispositions is radically changing healthcare. As much of chemotherapy is tailored to genetics, it may also be crucial to consider this in consideration for potential medication side effects. While it is tempting to consider chemotherapy-induced neuropathy as a single entity, it may be more pragmatic and useful to consider each neuropathy separately as a specific side effect of a specific drug class, as successful prevention and/or treatment may differ. The paucity of beneficial treatments (and preventive measures) of CIPN makes the understanding of the underlying pathophysiological processes of CIPN an imperative on the road to the development of more targeted treatment options.
